# An Investigation of the Endocrine-Disruptive Effects of Bisphenol A in Human and Rat Fetal Testes

**DOI:** 10.1371/journal.pone.0117226

**Published:** 2015-02-23

**Authors:** Millissia Ben Maamar, Laurianne Lesné, Christèle Desdoits-Lethimonier, Isabelle Coiffec, Julie Lassurguère, Vincent Lavoué, Yoann Deceuninck, Jean-Philippe Antignac, Bruno Le Bizec, Elisabeth Perdu, Daniel Zalko, Charles Pineau, Cécile Chevrier, Nathalie Dejucq-Rainsford, Séverine Mazaud-Guittot, Bernard Jégou

**Affiliations:** 1 Inserm (Institut national de la santé et de la recherche médicale), IRSET, U1085, SFR Biosit, Campus de Beaulieu, Rennes, CEDEX, France; 2 Université de Rennes I, Campus de Beaulieu, Rennes, CEDEX, France; 3 CHU Rennes, Service Gynécologie et Obstétrique, Rennes, France; 4 USC 1329 LABERCA, ONIRIS, Atlanpôle—La Chantrerie, Nantes, France; 5 UMR 1331 TOXALIM, INRA (Institut National de la Recherche Agronomique), Chemin de Tournefeuille, Toulouse, France; 6 EHESP—School of Public Health, Avenue du Professeur Léon Bernard, Rennes, France; Universidad Miguel Hernández de Elche, SPAIN

## Abstract

Few studies have been undertaken to assess the possible effects of bisphenol A (BPA) on the reproductive hormone balance in animals or humans with often contradictory results. We investigated possible direct endocrine disruption by BPA of the fetal testes of 2 rat strains (14.5–17.5 days post-coitum) and humans (8–12 gestational weeks) and under different culture conditions. BPA concentrations of 10^-8^M and 10^-5^M for 72h reduced testosterone production by the Sprague-Dawley fetal rat testes, while only 10^-5^M suppressed it in the Wistar strain. The suppressive effects at 10^-5^M were seen as early as 24h and 48h in both strains. BPA at 10^-7^-10^-5^M for 72h suppressed the levels of fetal rat Leydig cell insulin-like factor 3 (INSL3). BPA exposure at 10^-8^M, 10^-7^M, and 10^-5^M for 72h inhibited testosterone production in fetal human testes. For the lowest doses, the effects observed occurred only when no gonadotrophin was added to the culture media and were associated with a poorly preserved testicular morphology. We concluded that (i) BPA can display anti-androgenic effects both in rat and human fetal testes; (ii) it is essential to ascertain that the divergent effects of endocrine disruptors between species *in vitro* do not result from the culture conditions used, and/or the rodent strain selected; (iii) the optimization of each *in vitro* assay for a given species should be a major objective rather than the search of an hypothetical trans-species consensual model-system, as the organization of the testis is intrinsically different between mammalian species; (iv) due to the uncertainty existing on the internal exposure of the human fetal testis to BPA, and the insufficient number of epidemiological studies on the endocrine disruptive effects of BPA, caution should be taken in the extrapolation of our present results to the human reproductive health after fetal exposure to BPA.

## Introduction

Of the thousands of anthropogenic chemicals present in the environment, those suspected of posing a threat to the health of the living organisms by disrupting their endocrine systems, referred to as endocrine-disrupting chemicals (EDCs), have attracted much attention over the past two decades. Among potential EDCs, bisphenol A (BPA) [2,2-bis (4-hydroxyphenyl) propane; CAS # 80 b5-7] is the object of great concern because of its widespread use, shown by the several million tons currently produced throughout the world each year [1]. BPA is ubiquitous in the environment, used primarily to manufacture polycarbonate plastic, or as a non-polymer additive to other plastics and to epoxy resins. It is therefore present in a vast range of products [2, 3] to which the general human population, from newborn babies to adults [4–6], is exposed. More than 5000 safety-related studies have been published on this molecule, but a fierce debate still rages as to whether and how BPA causes adverse effects in humans [7–11].

Other EDCs have drawn attention due to their potential effects in mammals on the development and functions of reproductive organs, which depends crucially on hormones. These include phthalates, which are also widely used, in the plastics and pesticide industries, among others (recently reviewed by Albert & Jégou 2014 [12]). In contrast, only a few studies have so far examined this issue for BPA by either toxicological or epidemiological assessments (recently reviewed by Rochester 2013 [13]). They have shown that BPA doses below the current reference exposure limits for humans can impair reproductive physiology in CF-1 mice ([14]; 2.4 μg/kg) and induce nonsocial behavior in Sprague-Dawley rats ([15]; 40 μg/kg/day). Exposure to BPA at concentrations considered environmentally relevant (2 ng/g or 20 ng/g of body weight) during the perinatal development of mice has been shown to impair testicular development at puberty and in adulthood [16, 17]. Exposure to BPA *in utero* inhibits the testosterone surge in the rat neonatal period [18]. BPA (10^-7^–10^-4^ mol/L) also alters testosterone production by mouse fetal testes *in vitro* [19]. However, *in utero* exposure of Sprague-Dawley rats to 40 mg/kg/day [20] or 500 mg/kg/day BPA [21] was not associated with any change in the anogenital distance (AGD), which reflects *in utero* exposure to androgens in male pups. By contrast, exposure of pregnant Wistar-Furth rats to 250 μg/kg/day did reduce the AGD in their male pups and thus reflected a deficiency in androgen production or action [22].

Epidemiological evidences are even scarcer with only a few studies of the possible consequence of *in utero* BPA exposure on male urogenital tract’s development. While neither cryptorchidism [23–25] nor hypospadias [26] has been associated with exposure to BPA or biphenolic compounds during gestation. Sons of parents occupationally exposed to BPA have shorter AGDs and this association is significant for maternal exposure [27]. This observation suggests that BPA can display anti-androgenic properties *in utero* in humans, a finding reinforced by a recent report that BPA at concentrations of 10^-8^ M, 10^-7^ M, and 10^-5^ M inhibits testosterone production by explants of human fetal testes in culture [28]. Yet, in that study and in contrast to what was observed with the human fetal testis explant cultures, only the relatively high concentration of 10^-5^M BPA significantly lowered the *in vitro* testosterone levels produced by rat and mice testes explants. This finding led these authors to conclude that “rodents are not relevant for predicting the effect of low BPA concentrations on the endocrine function of human fetal testis” [28].

In view of the very few studies available and especially the very negative potential consequences of the possible disqualification of rodents as pertinent species for assessing risks from BPA and BPA-related molecules, we investigated the extent to which BPA modifies hormone levels produced by human and rat fetal testes in different culture conditions. Potential differences between rat strains were also examined.

## Materials and Methods

### Ethic Statement

The animal facility used for the present study is licensed by the French Ministry of Agriculture (agreement # C 35-238-19). All the experimental procedures followed ethical principles according to the NIH Guide for Care and Use of the Laboratory Animals and were approved by the Rennes Animal Experimentation Ethics Committee (#R-2012-CCh-01).

First trimester human fetuses (8–12 gestational weeks (GW)) are obtained from pregnant women following legally induced abortion performed in Rennes Sud Hospital. The women received an information leaflet by the medical staff. No woman underage donated their aborted fetus to this research study. Abortion products were collected after a two-step procedure coupling a verbal preselection consent followed by a written consent from those who eventually agreed that their fetus would participate in the study, in accordance with national guidelines. The national agency for biomedical research (authorization #PFS09-011, “Etude de différenciation normale et pathologique des gonades humaines”; Agence de la Biomédecine) and the Local ethic comittee of Rennes hospital (advice # 11–48) approved the whole procedure.

### Sample collection

**Rat fetal testis**. Sprague-Dawley and Wistar pregnant dams (Janvier Labs, Le Genest Saint-Isle, France) were anesthetized on gestational day 14.5 (GD 14.5) by intraperitoneal injection of 100 mg/kg ketamine (VIRBAC, Carros, France) and 10 mg/kg xylazine (Bayer Healthcare, Loos, France). Fetuses were dissected and testes removed under a binocular microscope.

**Human fetal testis**. First-trimester human fetal testes (7–12 GW) were obtained from pregnant adult women following legal terminations of pregnancy at Rennes University Hospital, France. No termination was motivated by fetal abnormality. The testes were recovered from the aspiration product under a binocular microscope (Olympus SZX7, Lille, France) and immediately placed in cold phosphate-buffered saline (PBS) as previously described [29].

### Culture procedures

**Rat fetal testis**. Immediately after explanting, each GD 14.5 testis with its adjacent mesonephros was placed on an 0.45 μM filter (Millipore, Billerica, MA) floating on 0.4 mL of M199 medium (Life Technologies SAS, Villebon sur Yvette, France) in 24-well plates incubated for 72 hours with the medium refreshed every day, as previously described [30]. The solutions were prepared for final BPA (Sigma-Aldrich, Saint-Quentin, France) concentrations of 10^-9^ M to 10^-5^ M. Dimethyl sulfoxide (DMSO; Sigma-Aldrich) was used as a vehicle in the experiments involving both Sprague-Dawley and Wistar fetal testes, or ethanol for the Sprague-Dawley fetal testes only. We added 0.1% of DMSO or 0.1% ethanol to control media. The medium from each culture was collected every day and stored at -80°C. After 72 h, testes were fixed in Bouin’s fluid for 90 min, dehydrated, and embedded in paraffin.

**Human fetal testis**. The testes recovered were cut into explants smaller than 1 mm^3^ as deduced from N’Tumba-Byn et al. [28]. For the younger testes (7–9.86 GW), 4 wells for 4 different culture conditions (*i*.*e.*, 1 control and 3 BPA concentrations) were designed, and for the older testes (10–12 GW), 5 wells for 5 different culture conditions (*i*.*e.*, 1 control and 4 BPA concentrations). For the hCG condition, the testicular explants were cultured in cell-culture inserts (0.4 μm pores, Becton-Dickinson, Le Pont de Claix, France) placed in 24-well companion plates (Becton-Dickinson, Le Pont de Claix, France), in strict accordance with a method previously described [29]. For the hLH or no gonadotrophin conditions, the testicular explants were cultured in cell culture inserts (0.4-μm pores; Millipore, Billerica, MA) placed in 24-well companion plates (Becton-Dickinson, Le Pont de Claix, France) according to N’Tumba-Byn et al. [28]. Some cultures used no gonadotrophin supplementation, others hCG (Sigma Aldrich Chemicals) added to a final concentration of 0.1 IU/mL [31], and still others hLH (Sigma Aldrich Chemicals) at a final concentration of 0.1 μg/mL [32]. In some cases, both testes of a single fetus were cultivated according to an identical experimental plan, but one testis was exposed to a gonadotropin and not the contralateral one. Cultures were incubated at 37°C for 96 h under a humidified atmosphere of 95% air and 5% CO2. The medium was removed every 24 h and divided into at least 2 aliquots that were immediately snap-frozen on dry ice and stored at -80°C. After the first 24 h of culture (D0), the explants were exposed to BPA treatment by adding concentrations of 10^-5^ M, 10^-6^ M, 10^-7^ M, and 10^-8^ M to the medium to assess dose-response effects. The possible influence of the solvent used to dissolve BPA—DMSO or ethanol both at a final concentration of 0.1% — was also assessed.

### BPA concentrations in the medium samples

To assess the actual concentration of BPA to which the explants were exposed, the following samples were considered: control ethanol and DMSO, BPA 10^-8^ M diluted in ethanol and DMSO and BPA 10^-5^M diluted in ethanol and DMSO medium were incubated for 72 h in the same conditions as described in the culture procedures section but without any fetal testis explants.

The standard operating procedure consisted in adding 100 μL of medium to an Eppendorf tube. Each sample was then fortified with 1×10^-6^ M of internal standard (^13^C-BPA) vortexed for 30 sec and left in contact with the internal standard for 1 h. Afterwards, 300 μL of ethyl acetate was added. The sample was shaken for around 30 sec and centrifuged at 4000 rpm for 15 min at 4°C. The organic phase was collected using a Pasteur pipette, transferred to a gas chromatography vial, and evaporated to dryness under a gentle nitrogen stream (45°C). 20 μL of MSTFA [N-Methyl-N-(trimethylsilyl)trifluoroacetamide] were added to the dry residue and the vial was heated for 30 min at 45°C. Detection was achieved using gas chromatography coupled to tandem mass spectrometry (GC-MS/MS), in the selected reaction monitoring (SRM) mode as previously described [33].

The control of in-laboratory environmental contamination is a major critical issue with regard to the analyses of such ubiquitous contaminant. In the present case, the in-laboratory environmental background contamination was evaluated for each batch of characterized samples by analyzing at least 6 blank samples and calculating the corresponding average level and related variability of the monitored procedural background signal. In addition, several quality control dispositions were implemented: the standard operating procedure was applied in a positive-pressure room, Eppendorf tube used were previously washed with 200 μL of methanol and glassware (Pasteur pipettes and GC vials) used in the extraction procedure was previously heated for 12h at 500°C.

The background BPA contamination in our ethanol control medium samples was 3.7±0.6×10^-8^ M BPA. The BPA measured in the 10^-8^ M solution in ethanol was 8.2±3.6×10^-8^ M. The actual concentration of BPA at 10^-5^ M was 3.5±1.3×10^-5^ M. In DMSO, there were no or only slight differences in the actual BPA concentrations between the 3 prepared solutions and the measured BPA concentrations: control DMSO (1×10^-8^ M), 10^-8^ M DMSO (measured 1.5×10^-8^M) and 10^-5^ M DMSO (measured 0.9×10^-5^ M). This demonstrates that the concentrations of BPA present in the solutions that we prepared were somewhat consistently higher than expected when ethanol was used instead of DMSO. This is likely to result either from a contamination of the ethanol used to dilute BPA, or from the fact that ethanol has the ability to fix or to extract some environmental BPA during the experimental procedures undertaken in this study. Despite these differences between the BPA concentrations that we prepared and those that we measured, the solutions in ethanol are described hereafter as the control ethanol, 10^-8^ M ethanol, and 10^-5^ M ethanol, for clarity’s sake.

### Immunostaining and stereology

To assess the possible effects of BPA on the morphological integrity of the rat testes, immunohistochemistry was performed on Bouin solution-fixed, paraffin-embedded tissues. In the fetal rat testes, the different cell types were identified by immunohistochemistry and counted with the Computer-Assisted Stereology Toolbox (CAST) Grid system (Olympus, Copenhagen, Denmark) [34]. Sertoli cells were stained with goat primary antibody against GATA-4 (1:100; Santa Cruz Biotechnology, CA, USA), and Leydig cells with rabbit primary antibody against 3-beta hydroxysteroid deshydrogenase (HSD3B) (1:500; provided by J.J. Feige, Inserm U1036, Grenoble, France). Gonocytes were identified after hematoxylin and eosin staining. The gonocyte and Sertoli cell mitotic indexes were assessed by adding 50 μM of BrdU, in the culture medium for the last 3 h of incubation. BrdU incorporation into cellular DNA was detected with a cell proliferation kit (Amersham, GE Healthcare, Buckinghamshire, UK), as previously described [30]. A cleaved-caspase 3 antibody (1/100; Cell Signaling, Ozyme, Saint Quentin en Yvelines, France) was used to count the testicular apoptotic cells as described in Desdoits-Lethimonier et al. 2012 [35].

In human fetal testes, the steroidogenic Leydig cells were labeled with rabbit anti- cytochrome P450, family 11, subfamily A, polypeptide 1 (CYP11A1) antibody (1/250; Sigma Aldrich). Three different observers independently blinded-assessed the integrity of the testes sections after the different cultures were completed.

### BPA biotransformation

To investigate the possible daily biotransformation capabilities of the fetal testes, we applied the same procedures described in the culture procedures section for Sprague-Dawley and human fetal testes; both were incubated with [^3^H]-BPA (1.66 KBq per well) adjusted with unlabeled BPA (Sigma) in 5 μL ethanol to reach the required dose of 0.1 μM. At the end of the incubation time, the media were collected from each culture in glass vials. Organs were collected separately in ethanol. Wells and inserts were rinsed with 0.5 mL ethanol. [^3^H]-BPA, with a specific activity of 185 GBq/mmol, was purchased from Moravek Biochemicals (Brea, CA). Its radiopurity was verified by radio-HPLC and exceeded 99.9%. The radioactivity levels of the incubation media, ethanol organ extracts, and ethanol washings were measured from aliquots (25 μL) by liquid scintillation counting in a Packard Tri-Carb 1500 counter with Ultima Gold (Packard Instruments Co., Downers Grove, IL) as the scintillation cocktail. Aliquots of each medium of organ incubation were analyzed directly by radio-HPLC. Ethanolic extracts from organs were evaporated and dissolved in mobile phase A to be injected in the RP-HPLC system. Ultrapure water from the Milli-Q system (Millipore, Saint Quentin en Yvelines, France) was used for the HPLC mobile phases. The HPLC system was a Spectra Physics System (P1000) coupled, for radioactivity detection, to a Packard Flo-One A500 detector with Flo-Scint II as the scintillation cocktail (Packard Bioscience). The HPLC system consisted of a Zorbax C18 column (250 × 4.6 mm, 5 μm) (Interchim, Montluçon, France) coupled to a Kromasil C18 guard precolumn (18 × 4.6 mm, 5 μm) (Interchim). Analytical conditions were detailed previously in Audebert et al. 2011 [36] Metabolites were quantified by integrating the area under the peaks monitored by radioactivity detection. The structural characterization of BPA sulfate was achieved by retention time comparison with the authentic standard [37], and was confirmed by specific enzymatic hydrolysis assays carried out on incubation supernatants using Aerobacter aeruginosa sulfatase (Sigma), as previously described in Audebert et al.[36].

### Hormone assays

Testosterone was assayed with a specific radioimmunoassay (RIA; Immunotech, Beckman Coulter, Villepinte, France). The intra- and inter-assay coefficients of variation were ≤ 8.6% and 11.9%, respectively. A culture of rat fetal testis explants was considered successful when the basal concentration (without BPA) of testosterone measured in the medium was > 0.5 ng/mL after 72 h and increased throughout the experiment.

For the human fetal testes, day 0 (the first 24 hours, D0) served as the baseline value for normalization of hormone production per piece of testis after 24 h (D1), 48 h (D2) and 72 h (D3) of culture with BPA. Depending on fetal age, we estimated that normal relative testosterone production in the control experiments should be above 0.7 as calculated at the D3 normalized to the D0 testosterone production of the same culture well (Relative Testosterone Production (RTP)) for the 7–9.86 GW explants and ≥ 0.1 for the 10–12 GW explants. Specifically, at D0 (before addition of BPA), testes explanted at 7.0–7.9, 8.0–8.9, 9.0–9.9, 10.0–10.9 and 11.0–11.9 GW produced 2.3±1.09 ng/h (n = 10), 6.3±1.8 ng/h (n = 10), 14.3±1.06 ng/h (n = 13), 15.1±1.24 ng/h (n = 18) and 17.6±2.8 ng/h (n = 12) of testosterone, respectively. For the rat fetal testes, INSL3 levels were assessed with an enzyme-linked immunosorbent assay (ELISA) kit (USCN Life Science, Euromedex, Souffelweyersheim, France), with an intra-assay CV 10% and an inter-assay CV 12%. In control DMSO conditions, Sprague-Dawley fetal testes produced an average ± SEM of 174.9±39.5 pg/mL after 72 h of culture. For the human fetal testes, INSL3 was assayed with a commercial RIA (RK-035-27, Phoenix France, Strasbourg, France). The intra- and inter-assay coefficients of variation were ≤ 15% and 7%, respectively. Specifically, at D0 (before addition of BPA), testes explanted at 7.0–7.9, 8.0–8.9, 9.0–9.9, 10.0–10.9 and 11.0–11.9 GW produced 3.99±2.39 pg/h (n = 3), 6.95±1.9 pg/h (n = 4), 7.22±0.88 pg/h (n = 15), 17.4±6.34 pg/h (n = 20) and 19.3±4.1 pg/h (n = 8) of INSL3, respectively. Normal relative INSL3 production (RIP) should be > 1.0 for all fetuses. Results are expressed as normalized production of treated samples as the percentage of that of the respective untreated first day of culture sample (RIP) or as normalized production of treated samples as the percentage of that of the respective untreated first day of culture and control (RIP, %Ctrl).

### Statistical analysis

For the rat fetal testis, values are mean ± SEM. Analysis of variance (one-way ANOVA) followed by the appropriate post-hoc test was used to compare differences between BPA treatments, as specified in each figure legend. The effects of vehicle and strain were analyzed with two-way ANOVA. The cell countings were analyzed using the non-parametric Wilcoxon signed rank test. For the human fetal testis, values (means ± SEM) are expressed as percentages from the respective untreated first day of culture basal sample and control. Differences between BPA treatments were analyzed with the non-parametric Wilcoxon signed rank test. Analysis of variance (one-way or two-way ANOVA) followed by the appropriate post-hoc test was used to compare differences between conditions, as specified in each figure legend.

For both culture-models, a BPA dose-response relationship was assessed for testosterone and INSL3 measurements using a non-parametric Spearman correlation test or a Pearson correlation test, depending on the normal distribution or not of the data. Significance was defined as a confidence level of *p*≤0.05. Statistical analyses were performed with the SigmaPlot 12.0 software package (Systat Software Inc, San Jose, CA).

## Results

### BPA biotransformation in rat and human testis

Radio-HPLC analyses were carried out for all medium samples and all tissue extracts. With the rat fetal testes, only the parent BPA was recovered after 24 h of incubation, regardless of the dose. This finding indicates that no biotransformation of BPA took place. In contrast, in the human fetal testes, about 10% of the recovered radioactivity was in the form of one BPA sulfate conjugate; this organ thus showed a low but actual ability to biotransform BPA.

**Fig 1:**
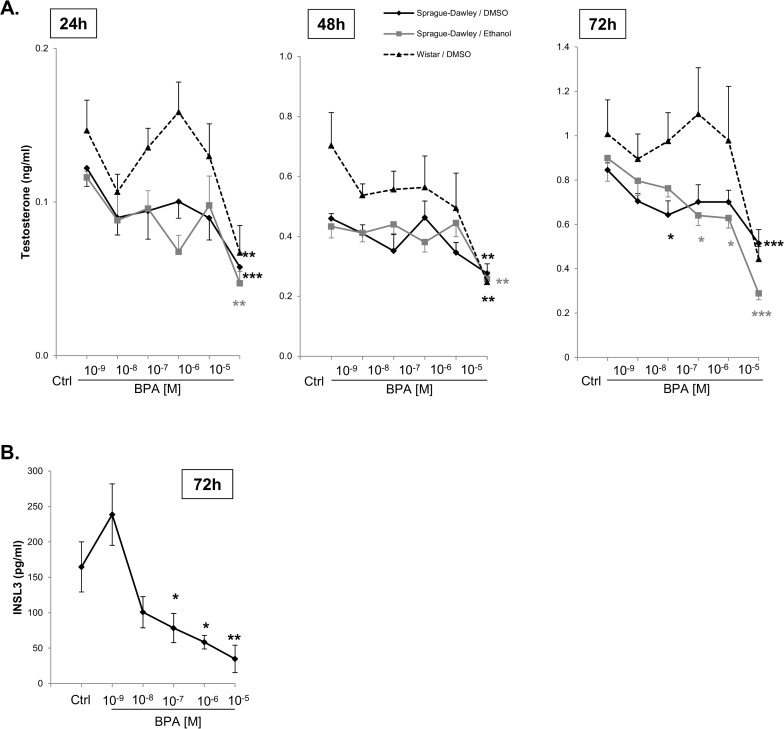
Time and dose-dependent effects of BPA (10^-9^M-10^-5^M) on the rat fetal testis. A) Basal testosterone production by the rat fetal testis: dose-dependent effects of BPA (10^-9^M-10^-5^M) on basal testosterone levels. Rat strain (Sprague-Dawley *versus* Wistar) and vehicle in which BPA solutions were tested and prepared (DMSO *versus* ethanol) were studied in order to determine their influence on the responses to BPA exposures. B) Dose-dependent effects of BPA (10^-9^M-10^-5^M) on the basal INSL3 production by the Sprague-Dawley fetal rat testis: BPA was diluted in DMSO and the media collected after 72hr of culture were assayed. Values are mean +/- SEM of 12–24 testes from at least 3 independent experiments. Dose-responses were analyzed for significance with one-way ANOVA, Holm-Sidak post-hoc test. The effects of vehicle and strain were analyzed with two-way ANOVA. *p <0.05, ** p <0.01, *** p <0.001.

**Rat fetal testis**. Testosterone levels produced by the Sprague-Dawley fetal testes in control cultures, as well as in the presence of BPA, did not significantly differ according to the solvent (DMSO or ethanol) used in the cultures (Fig. 1A). Testosterone production by these fetal testes was significantly inhibited by 10^-5^M BPA diluted in DMSO at all time periods (Fig. 1A; 24 h: -53%, p<0.001; 48 h: -40%, p<0.01; 72 h: -39%, p<0.001) and by 10^-8^M of BPA in DMSO after 72 h of culture (-24%, p<0.05) (Fig. 1A), and a dose-dependent suppression of testosterone was observed (p<0.001). Similarly, when the BPA was dissolved in ethanol, BPA 10^-5^ M ethanol also significantly inhibited testosterone production by these Sprague-Dawley testes after 24 and 48 h of culture (Fig. 1A: -56%, p<0.01 and -40%, p<0.01, respectively). After 72 h under the same conditions, dose-dependent suppression of testosterone was observed (p<0.001). A significant reduction was seen at 10^-7^M (-20%, p<0.05), 10^-6^M (-30%, p<0.05), and 10^-5^M (-68%, p<0.001) BPA diluted in ethanol (Fig. 1A).

In the presence of DMSO only, testosterone levels from the Wistar fetal testes were consistently significantly higher than those from Sprague-Dawley fetal testes at all time points investigated (Fig. 1A, 24 h: + 25%, 48 h + 52%, 72 h: +19%) in control cultures. They remained significantly higher than those in Sprague-Dawley fetal testes in the presence of 10^-9^–10^-6^M BPA diluted in DMSO (24 h: + 18–44%; 48 h: +21–58%; 72 h: +27–56%). Only the highest BPA concentration suppressed testosterone production by the Wistar testes at all time points (24 h: -54%, p<0.01; 48 h: -65%, p<0.01; 72 h:-56%, not significant) (Fig. 1A). After 72 h, no significant correlation between BPA concentrations and levels of INSL3 was found.

**Fig 2:**
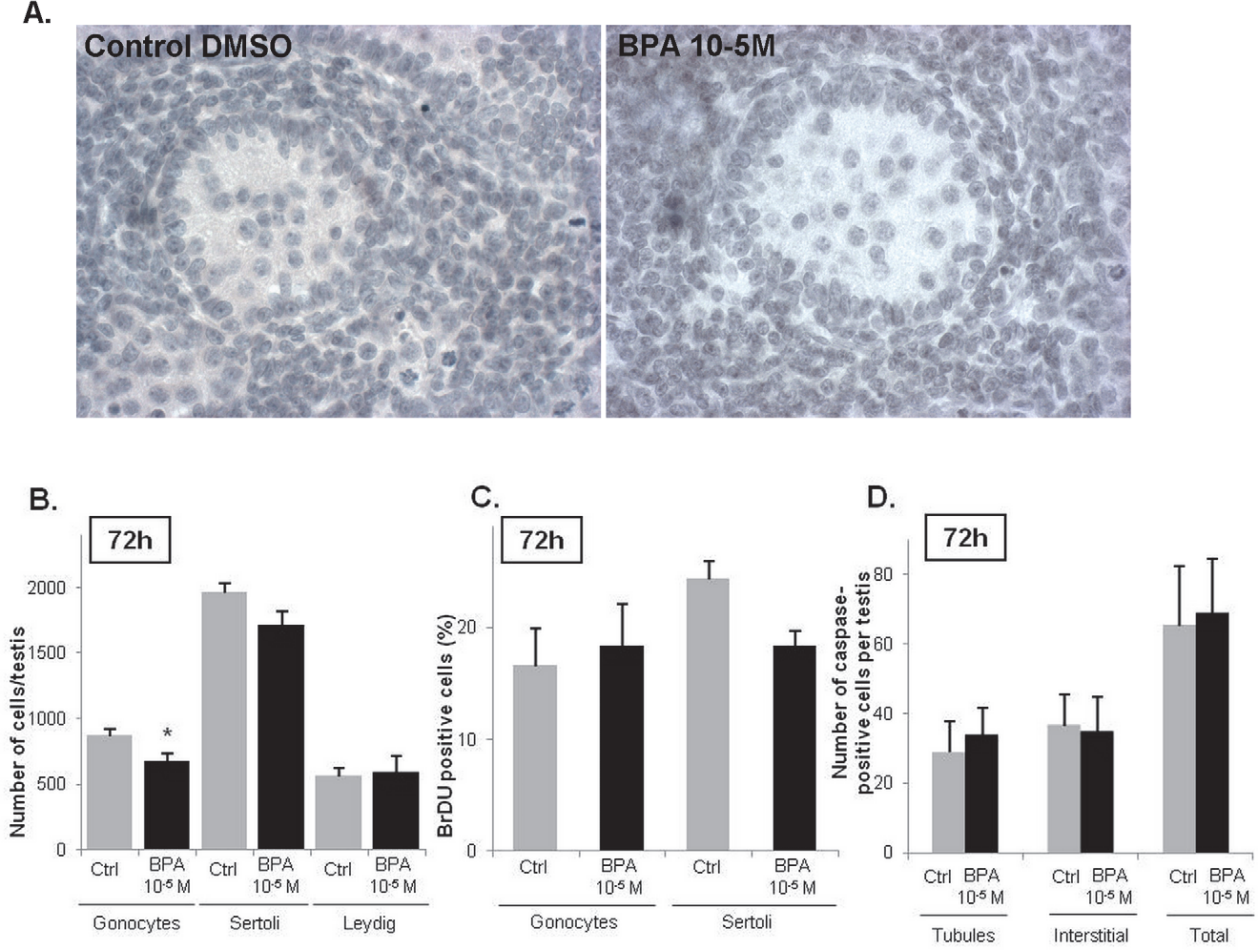
BPA effects at 10^-5^M on the testicular histology and testicular cells. A) A treatment with 10^-5^M of BPA did not affect the morphology and the testis organization of the fetal rat. B) Effect of 10^-5^M of BPA on the total number of gonocytes, Sertoli cells and Leydig cells. Values are mean ± SEM of 4–7 fetuses. Responses to BPA were measured by comparing one control testis (DMSO-treated) with the contralateral testis cultured in medium containing the tested factor. C) Quantitative analysis of BrdU incorporation into gonocytes and Sertoli cells after 72 h of culture measured as the percentage of BrdU-positive gonocytes or Sertoli cells in at least 1000 cells (*n = 4 fetuses*). D) Apoptosis was detected using a cleaved-caspase 3 staining, caspase-3 being cleaved in the cells undergoing apoptosis. Caspase 3-positive cells were counted on the whole testis with regard to their tubular or interstitial localization (*n = 5 fetuses)*. *p < 0.05 by Wilcoxon signed rank tests on paired data.

After fetal testes from Sprague-Dawley rats were cultured for 72 h with BPA in DMSO, INSL3 production by fetal Leydig cells was lower than in control cultures from the dose of 10^-8^M (-42%); this inhibition was significant from 10^-7^M (-55%, p = 0.05) onwards: 10^-6^M: -67%, p<0.05; 10^-5^M: -76%, p<0.01 (Fig. 1B). None of the BPA treatments at any time point had any apparent effect on the gross morphology of the rat fetal testis (Fig. 2A). After Sprague-Dawley fetal testes were cultured for 72 h with 10^-5^M BPA diluted in DMSO, the number of gonocytes decreased (Fig. 2B: -23%, p<0.05). The number of BrdU-labeled gonocytes was not affected by this BPA concentration (Fig. 2C). Under the same culture conditions, neither the number of Sertoli cells (Fig. 2B) nor the number of BrdU-labeled Sertoli cells was significantly affected by the treatment (Fig. 2C). In the presence of 10^-5^M BPA, the number of Leydig cells was not affected (Fig. 2B). Both intratubular and interstitial compartments were checked for apoptosis, and neither was significantly affected by the BPA treatment at 10^-5^M, the highest concentration to which the rat fetal testes were exposed (Fig. 2D).

**Fig 3:**
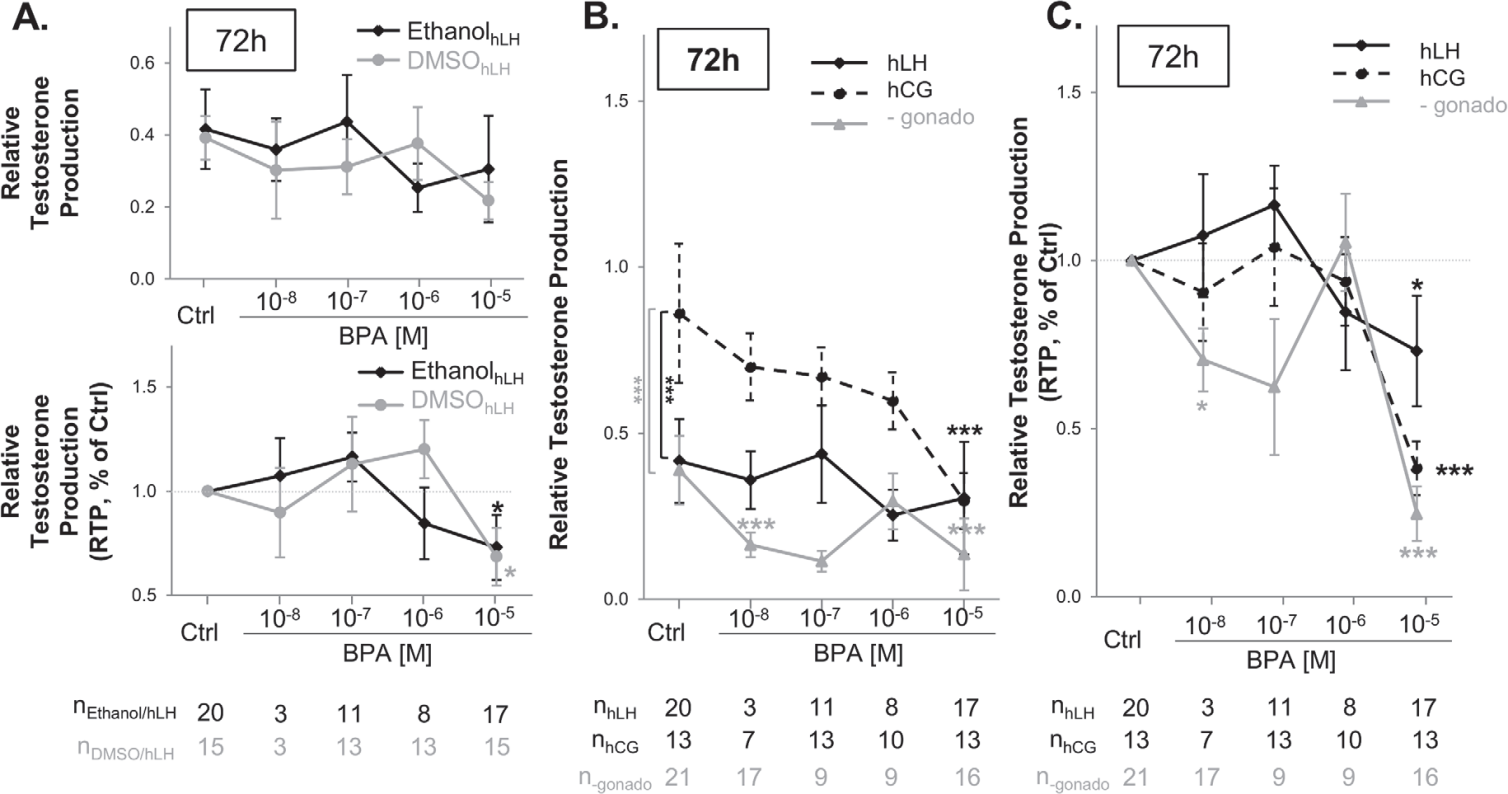
Effects of BPA on testosterone production after 72h of culture of 7–12 GW human fetal testis explants. A) Dose-dependent effects of BPA—diluted in DMSO or ethanol—with hLH on testosterone production by human fetal testis explants (RTP; RTP, %Ctrl): BPA was diluted in DMSO or ethanol and the media collected after 72 hr of culture were assayed. Results are expressed as normalized production of testosterone of treated samples as the percentage of that of the respective untreated first day of culture sample (RTP) (top) and as the percentage of that of the respective untreated first day of culture sample and control (RTP, %Ctrl) (bottom). Values are mean +/- SEM of testosterone from the respective untreated first day of culture basal sample and control. The number of testes (n) is indicated below the graphic for each condition. Dose-responses were analyzed for significance with a Wilcoxon test. The effects of the vehicle were analyzed with two-way ANOVA. *p<0.05. B) Testosterone production after culture of human fetal testis in the presence of BPA and hCG, hLH or no gonadotrophin (-gonado) supplementing the medium (RTP). n = 7–13 testes for the hCG group, n = 3–20 testes for hLH group and n = 9–21 in the absence of gonadotrophin. *p<0.05, ***p<0.001 (two-way ANOVA followed by a Holm-Sidak test or a Wilcoxon test to compare matched samples). C) Testosterone production represented as a fold change from the respective first day of culture sample and control (RTP, %Ctrl). Results are expressed as normalized production of testosterone of treated samples as the percentage of that of the respective untreated first day of culture sample (RTP) and control (RTP, %Ctrl). n = 7–13 testes for the hCG group, n = 3–20 testes for hLH group and n = 9–21 in the absence of gonadotrophin. *p<0.05, ***p<0.001 (two-way ANOVA followed by a Holm-Sidak test or a Wilcoxon test to compare matched samples).

**Human fetal testis**. As described above in rats, human fetal testosterone levels did not differ significantly whether the testes were cultured with DMSO or ethanol in control conditions (Fig. 3A). BPA, whether diluted in DMSO or ethanol, did not significantly change testosterone production levels (Relative Testosterone Production, or RTP) at any concentration (Fig. 3A: p = 0.404). The same pattern of BPA-induced effects on testosterone was observed when the data were expressed as testosterone production normalized to the D0 of the same well and to the control (RTP, %Ctrl, p = 0.206). BPA at 10^-5^M induced a significant decrease of the RTP, %Ctrl whatever the solvent (Fig. 3A: -28% when diluted in DMSO, p<0.05; -35% in ethanol, p<0.05).

When hCG was added to culture media, testosterone levels were much higher (p<0.001) than when hLH was present or when there was no gonadotrophin of any kind (Fig. 3B). Under control conditions, whether or not gonadotrophin was present, testosterone levels declined between the first and the last day of culture (Fig. 3B: about 60%). In sharp contrast, when hCG was used, testosterone only marginally decreased (Fig. 3B: -10%). In the presence of hCG, BPA induced a progressive decline of testosterone, significant at 10^-5^ M compared to the control (Fig. 3B: -60%, p<0.001). A statistically significant correlation was observed between a decreasing production of testosterone and an increasing concentration of BPA (Fig. 3B: p<0.01). Furthermore, when the medium was supplemented with hLH, BPA inhibited testosterone production at 10^-6^M (Fig. 3B: -29.8%, p = 0.059). Only when no gonadotrophin at all was added did BPA significantly suppress testosterone at the lowest dose of 10^-8^ M (Fig. 3B: -23%, p<0.001), which was actually closer to 10^-7^M in ethanol as measured under our no gonadotrophin conditions (see above) and at 10^-5^M (Fig. 3B: -26%, p<0.001). It also decreased the testosterone level by about 71% at 10^-7^ M (Fig. 3B: p = 0.055).

The same pattern of BPA-induced effects on testosterone was observed whether the data were expressed as testosterone production normalized to the D0 of the same well (RTP) or testosterone production normalized to the D0 of the same well and to the control (RTP, %Ctrl) (Fig. 3C). Thus, in the absence of gonadotrophin, BPA induced a decrease in testosterone levels of 30% at 10^-8^ M (p<0.05), 37.7% at 10^-7^ M (p = 0.148), and 76% at 10^-5^ M (p<0.001). Finally, with hLH, BPA inhibited testosterone production at a concentration of 10^-5^ M (Fig. 3C: -30%, p<0.05).

**Fig 4:**
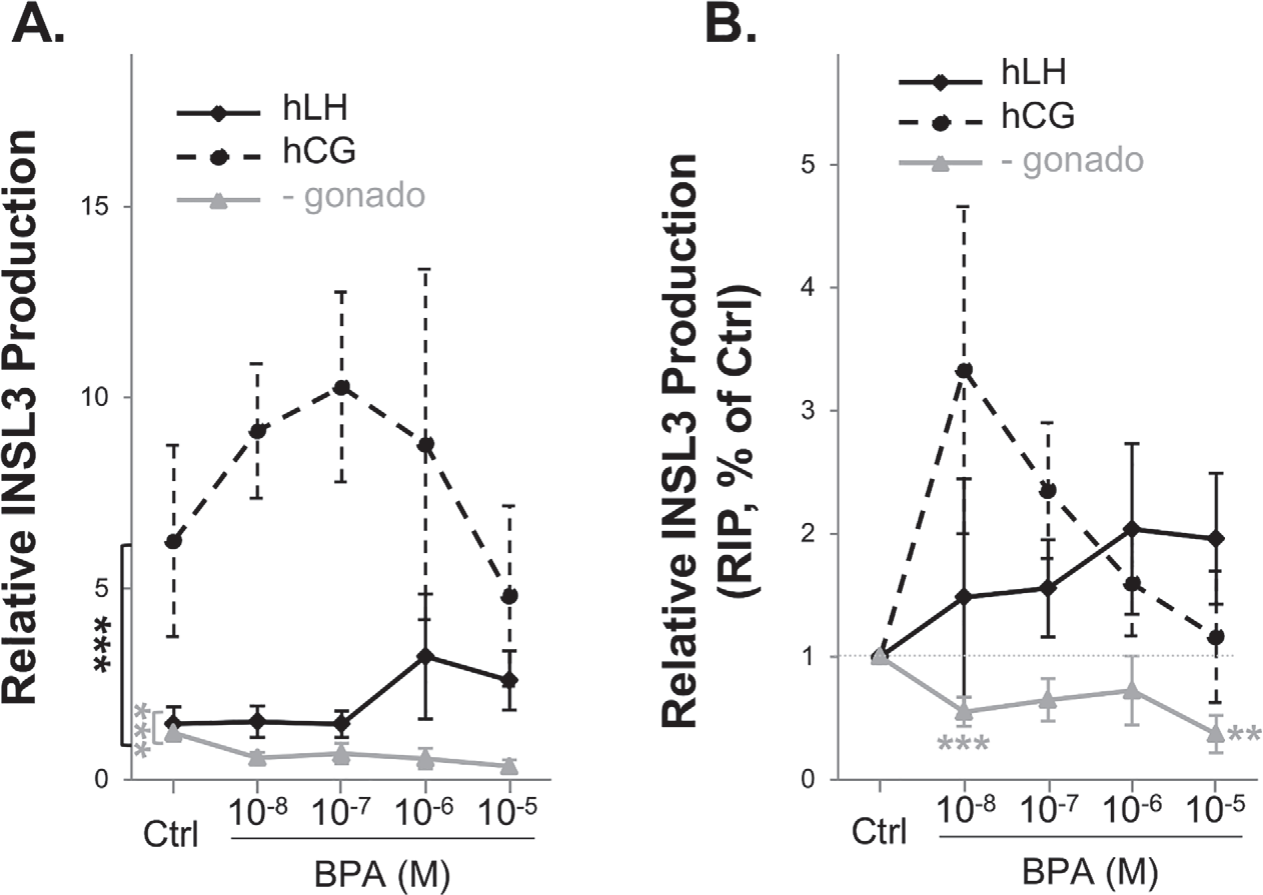
INSL3 production after culture of 8–12 GW human fetal testis explants in absence (Ctrl) or presence of 10^-8^M-10^-5^M. A) INSL3 production after culture of human fetal testis explants in the presence of BPA and hCG, hLH or no gonadotrophin (-gonado) supplementing the medium (RIP). INSL3 concentrations after culture of 8–12 GW human fetal testis in the presence of ethanol (Ctrl) or from 10^-8^–10^-5^ M BPA. Results are expressed as normalized production of INSL3 of treated samples as the percentage of that of the respective untreated first day of culture sample (RIP). n = 3–9 for hLH group, n = 3–11 testes for hCG group and n = 6–9 for the group without gonadotrophin. Values are means +/- SEM. *p<0.05, **p<0.01, ***p<0.001 (two-way ANOVA, followed by a Holm-Sidak test or a Wilcoxon test to compare matched sample). B) INSL3 production represented as normalized production of INSL3 of treated samples as the percentage of that of the respective untreated first day of culture sample and control (RIP, %Ctrl). INSL3 concentrations after culture of 8–12 GW human fetal testis explants in the presence of ethanol (Ctrl) or from 10^-8^–10^-5^ M BPA. Results are expressed as normalized production of INSL3 of treated samples as the percentage of that of the respective untreated first day of culture sample and control (RIP, %Ctrl). n = 3–9 for hLH group, n = 3–11 for hCG group and n = 6–9 for the group without gonadotrophin. Values are expressed as least squares means +/- SE. *p<0.05, **p<0.01, ***p<0.001 (two-way ANOVA, followed by a Holm-Sidak test or a Wilcoxon test to compare matched sample).

INSL3 production by human fetal testis explants was strongly higher in control cultures with hCG compared to those without hCG and to those with LH (Fig. 4A). Though not as high as with hCG, it was nevertheless always higher in the presence of hLH than in no gonadotrophin conditions (Fig. 4A). The relative production from D0 showed no suppression of INSL3 production by BPA for any condition (Fig. 4A). Compared with the normalized production in the controls over 3 days, 72 h of exposure to BPA at concentrations of 10^-8^ to 10^-5^ M did not modify INSL3 production in cultures supplemented with either hCG or hLH. However, when no gonadotrophin was used, BPA suppressed INSL3 levels, compared with control, at 10^-8^ M (Fig. 4B: -53%, p<0.05) and 10^-5^ M (Fig. 4B: -71%, p<0.001).

Regardless of the concentration of BPA used, Sertoli cell production of AMH did not change (data not shown).

**Fig 5:**
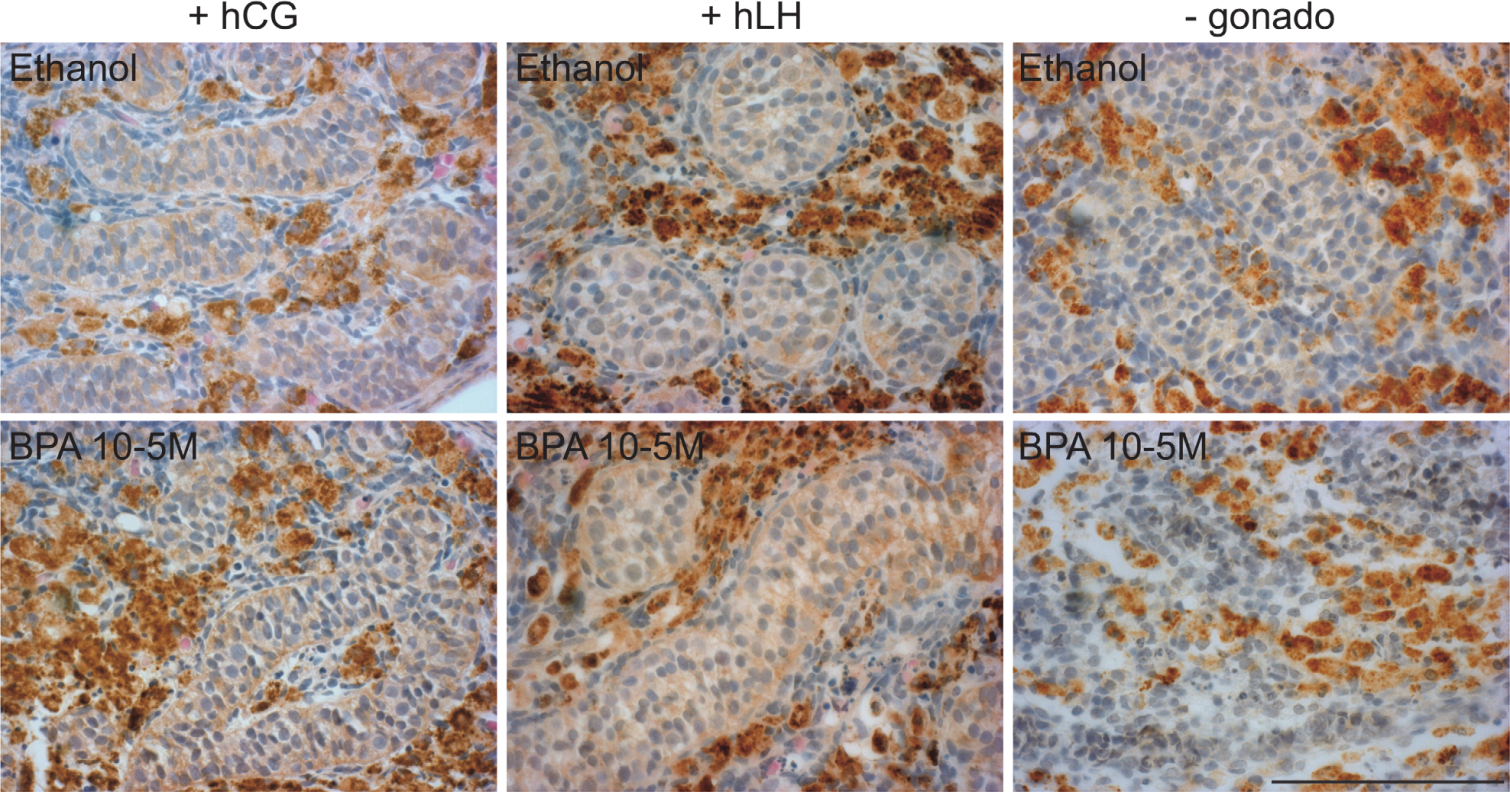
Representative immunostaining of CYP11A1 of human fetal testis explants 11–12 GW. The 3,3-diaminobenzidine tetrahydrochloride staining appears dark brown in all panels, and sections were counterstained with hematoxylin. Testis cords could be easily delineated in all treated explants except in the sections of explants that have been exposed to BPA 10^-5^M without gonadotrophin, in which the boundaries of the testicular cords appeared somewhat blunted. Scale bar corresponds to 100 μm.

The suppressive effect of BPA on testosterone was not associated with any effect on the gross morphology of the human fetal testis explants when hCG or hLH was present in the culture (Fig. 5). When no gonadotrophin was used, however, the boundaries of the sexual cords notably appeared to be much less visible after exposure to 10^-5^M of BPA: the testicular tissue appeared looser, leaving frequently small spaces getting apparent over the tissue sections (Fig. 5).

## Discussion

Because it is impossible for obvious ethical reasons to assess the effects of BPA on the development and function of human fetal testes *in vivo*, various experiments using *in vitro* testes are required to investigate the effects of potential EDCs [12].

Gestational exposure to BPA is suspected to increase miscarriage rates and induce abnormalities during gestation and in birth outcomes such as low birth weight, altered behavior in children or childhood asthma (recently reviewed by Rochester 2013 [13]). Nonetheless, toxicological studies in animals indicate that BPA can exert endocrine disruptive effects [16–22, 38, 39] and only one epidemiological study in humans suggests that BPA might exert anti-androgenic effects on the fetus [27].

In view of the very small number of reprotoxicological and epidemiological studies and their frequent contradictory findings, the recent study using human fetal testes [28] has drawn attention. It concluded that, in contrast to the situation observed in rats and mice, BPA has “a deleterious effect […] on fetal Leydig cells function in human for concentrations from 10^-8^ M upwards” and therefore that “species-specific differences raise concerns about extrapolation of data from rodent studies to human risk assessment”, to the point that these “findings challenge the widespread use of rodent models to assess the toxic effects of EDCs”. In view of the importance of these statements for the risk assessment of BPA and, more generally, of EDCs (should we have to rely only on human abortion products in culture for the risk assessment?), we conducted the series of experiments presented here, necessary for re-examining the effects of BPA on fetal testes from humans and rats. Our main goal was to ensure that the relative sensitivity of the human tissue to the anti-androgenic effects of BPA *in vitro*, compared with the relative unresponsiveness of the rat tissue was indeed due to interspecies differences and not to culture design and conditions.

Compared with rodent experiments, the use of human fetal testes *in vitro* imposes unavoidable constraints. The most important is that because of the rarity of the material and the small size of the fetal testes, this gonad tissue must be fragmented into small explants, so that each testis can be its own control, with some explants of a given testis serving as controls for the others. As a consequence, the data for the human fetal testis must be expressed as either normalized production from the first day of culture (RTP for testosterone and RIP for INSL3; see figure legends) or as normalized production from the first day of culture compared to that of control (RTP or RIP, % of control; see figure legends). Another consequence of the rarity and intrinsic constraints in experimentations with human fetal testes, relatively high experimental variations occurred. To take into account these variations, we have used up to 89 testes from 60 abortion procedures. As indicated before, experimental variations have to be present in mind when designing *ex vivo* or xenograft experiments using human testes (Albert and Jégou 2014)[12]. In contrast to the situation in humans, there is no such limitation in the availability of rat fetal testes; the ability to isolate and culture them compensates for their small size. The rat fetal culture system that we use here, previously named FEGA (for FEtal Gonad Assay), is based on the use of Sprague-Dawley rats and was previously optimized in studies to assess the effects of diethylstilbestrol (DES), estradiol, phthalates, and analgesics [30, 34, 40–42]. In confirmation of a previous *in utero* study/finding [43], the Wistar rat fetal testes, which we used here for the first time, produced much higher levels of testosterone *in vitro* than observed for Sprague-Dawley rats. However, while BPA significantly inhibited testosterone production at 10^-8^ M-10^-5^ M in the Sprague-Dawley fetal testes we used, only the 10^-5^ M dose significantly suppressed testosterone in the Wistar rats. This is likely to explain at least in part why BPA 10^-5^ M was the only effective dose that inhibited testosterone in the study of N’Tumba-Byn et al. [28], for they used Wistar rats for their experiments. Some of their other methodological options, in particular, the handicaps imposed by their choice to adjust the conditions of the rat and mouse *in vitro* tests to the constraints of the human test, most probably contribute to the poor sensitivity to BPA that they observed in the rodent culture systems.

We also show here that BPA inhibited INSL3 production dose-dependently in our rat experiments. This demonstrates that the BPA’s endocrine-disruptive effects on rat Leydig cells are not exclusive to steroidogenesis. They thus differ from the anti-androgenic effects observed for analgesics, which have previously been shown to be steroid-specific [42]. We also demonstrate that the effects of BPA on rat Leydig cells occurred independently of any change in the number of these cells and of any alteration of Sertoli cell number and function. Only rat gonocytes declined in numbers with BPA exposure but the mitotic index of the germ cells was not affected. This reduction in gonocytes was probably too modest to generate a significant increase in the actual number of apoptotic germ cells within the seminiferous cords. Alternatively, the kinetics of germ cell disappearance may have been too fast to allow an increase in the number of apoptotic cells to be recognized. This deleterious effect on the number of germ cells has previously been shown to occur when human fetal testes were exposed *in vitro* to phthalates [44].

Because we have always used DMSO to dissolve the EDCs screened in our previous studies [30, 34, 40–42] while N’Tumba-Byn et al. [28] used ethanol as the vehicle for BPA, we also investigated whether the choice of solvent might interfere with the sensitivity of rat fetal testes to BPA. We demonstrated that the nature of the solvent had no or only marginal effects on either basal testosterone production or Leydig cell response to BPA in both rats and humans. This finding contrasts with the results of Dhooge et al. [45], who used a yeast estrogen screen assay and found marked differences in the incubation times required to elicit responses when they compared DMSO and ethanol as vehicles for dissolving the estrogenic compounds of interest.

Most importantly, our study reveals that the effects of BPA on testosterone production by human fetal testes depended dramatically on the presence of a gonadotrophin in the culture medium. Thus, the explants were markedly more sensitive to BPA when they were cultured without hCG or hLH: testosterone production by the testes decreased significantly at 10^-8^ M, 10^-7^ M, and 10^-5^ M instead of only at 10^-5^ M when hCG or hLH were present in the culture media. Our results demonstrate that the most effective gonadotrophin for maintaining high testosterone levels was hCG. This is why we always used hCG (Mazaud-Guittot et al.[29] and here) instead of hLH like others [32]. This is logical given that testosterone regulation is driven by hCG during fetal development and before 15–20 GW, until control is eventually shifted to the fetal pituitary gland and LH secretion takes over control of testosterone production from hCG (reviewed by Huhtaniemi 1996[46]). To which extent the greater efficiency of hCG to stimulate and maintain testosterone levels would be linked to the longer half-life of this hormone when compared to LH is not known as the half-lives of these hormones have not been studied *ex vivo*. It is well known that LH and hCG share a common receptor and that hCG as a greater receptor binding affinity than LH which results in a greater biological activity (reviewed in Choi and Smitz 2014)[47]. Importantly, in rats during the corresponding phase of testicular development, contrary to humans, testosterone production is LH receptor-independent [48]. These points lead us to believe that the intrinsic differences in the morphology and physiology of humans and rats must be taken into consideration in the design of any experiments. The fact that concentrations as low as a theoretical concentration of 10^-8^ M can inhibit human fetal testosterone production confirms the results of N’Tumba-Byn et al. [28] which were obtained in the absence of gonadotrophins in the culture media. Nonetheless, we note that the BPA-induced inhibitory effects observed with the doses of 10^-8^M and 10^-7^M occurred when the morphology of the fetal testis explants was altered. This result casts doubts on whether the testosterone suppression observed here and in the N’Tumba-Byn study, at the lowest dose of BPA and in the absence of hCG actually results from any intrinsic anti-androgenicity of BPA. Instead, it may be that processing human fetal testes into small explants and culturing them in the absence of gonadotrophin renders them more vulnerable to toxic stress in general and in particular to BPA or to the mix of BPA and ethanol here. Moreover, we also observed differences between the BPA concentrations that were calculated and the concentrations actually measured in the media used for the experiments, the lowest concentration that we calculated being in fact higher than expected. These differences are probably due to the widespread contamination of the environment by BPA. This emphasizes the intrinsic difficulty to deal with the lowest BPA concentrations in the routine context of the laboratory.

Of note is that, in contrast to DES, phthalates and analgesics which showed anti-androgenicity in the fetal testis of rats but not in humans [29–31, 34, 42, 44], BPA is the only substance thus far identified as able to inhibit testosterone production both in the rat and human fetal testis. Note that the ability of BPA to suppress testosterone production has also been established in the human NCI-295R cell line [49], in human and rat testicular microsomes [50] and in different animal experiments [16–18].

We also demonstrate here that hCG is the gonadotrophin most effective in maintaining/stimulating human INSL3 levels. The relations between hCG and INSL3 remain to be fully clarified. Ivell et al. [51] recently reported that “LH or hCG have a markedly stimulatory effect on INSL3 production because the gonadotrophins can induce both Leydig cell proliferation and augment differentiation, and hence increase INSL3 production”. Further argument supporting a stimulatory effect of hCG on INSL3 in humans comes from the observation that patients with hypogonadotropic hypogonadism have an INSL3 production which is dependent on the differentiating effect of LH/hCG on Leydig cells [52]. While BPA had no effect on INSL3 produced by the human fetal testis explants cultured in the presence of gonadotrophin, the production of this Leydig cell hormone decreased in the absence of gonadotrophin. This finding is in line with the reduction of the steady-state levels of INSL3 mRNA after RT-PCR reported by N’Tumba-Byn et al. [28]. However, great caution should be taken in the interpretation of these results as the decrease in INSL3 levels seen here appeared only in the absence of gonadotrophins that is, in culture conditions that did not preserve testicular morphology.

In 1993, the U.S. Environmental Protection Agency set a reference dose of 50 μg/kg/day, which is presumed to be 1/1000 of the dose that exerts the lowest observable adverse effect (LOAEL; 50 mg/kg/day). However, there is still much incertitude and debate about the actual internal exposure to BPA in humans [4, 5, 13, 53–55]. According to Schonfelder et al. [56], blood BPA levels in a group of pregnant women ranged from 1.31×10^-9^ to 8.28×10^-8^ M, and in the cord blood from 8.76×10^-10^ to 4.03×10^-8^ M. Circulating levels of BPA have also been found to be correlated in pregnant women and their fetuses [56–60], and in the amniotic fluid [57]. Vandenberg et al. [53] calculated average circulating levels of BPA at 17×10^-9^M in pregnant women. Accordingly, values on the order of 10^-9^M have been detected in human amniotic fluid and cord blood [24]. Furthermore, several studies have established that BPA can cross the human placental barrier [56, 61, 62]. The correlation of BPA levels in both maternal and fetal blood strongly suggests that human fetuses are not *a priori* protected from exposure to BPA [60]. The results presented here demonstrate for the first time that the human fetal testis possesses the ability to biotransform BPA in the form of a sulfonate conjugate, although no evidence was found for the formation of BPA glucuronide, a major metabolite of BPA *in vivo*. Both conjugates are not active estrogens, contrary to parent BPA. Consequently, this strongly suggests that human fetal testes can to some extent protect themselves against BPA that reaches the fetus. According to Teeguarden et al. [55], only a very small proportion of circulating BPA (1–2%) occurs in the form of free BPA; the latter’s plasma concentration is thus below 10^-10^M. We must note, however, that no one actually knows the actual levels of exposure to the human fetus during gestation, including the age range corresponding to the ages of the human testes collected here after abortion (8–12 weeks of gestation). Moreover, the possibility that BPA might accumulate in early fetuses cannot be ruled out [53]. In addition to these important uncertainties about the internal fetal exposure, we found here that ubiquitous contamination by BPA limited our ability to experiment to concentrations of BPA lower than slightly above 10^-8^M when DMSO was used as vehicle and nearly 10^-7^M when ethanol was chosen instead.

## Conclusion

These experiments lead us to the following main conclusions: (*i*) dose-dependent anti-androgenic effects of BPA can be observed by using rat and human fetal testes in vitro; (*ii*) the inhibitory effects of BPA on rat and human fetal Leydig cells are not limited to steroidogenesis but can also be demonstrated for INSL3; this further establishes the potential endocrine capabilities of BPA; (*iii*) rats can be used for the male reproductive risk assessment of BPA (and others EDCs) in humans as long as the rat and human gonad assays are each properly optimized; (*iv*) the effects of BPA exposure on the human fetal testes at the lowest doses are encountered only in experimental conditions that do not guarantee the preservation of the integrity of the testicular architecture; and (*v*) due to the great uncertainty persisting about the internal exposure of human fetuses to BPA, the insufficient number of epidemiological studies and the inconsistencies between them, and the present limitations of the toxicology model-systems, caution should be taken in any extrapolation of our results to the humans reproductive system during fetal development.
