# Correction: An Investigation of the Endocrine-Disruptive Effects of Bisphenol A in Human and Rat Fetal Testes

**DOI:** 10.1371/journal.pone.0128051

**Published:** 2015-05-04

**Authors:** The PLOS ONE Staff

The abbreviation of the first author’s name appears incorrectly in the citation. The correct abbreviation is: Ben Maamar M. The correct citation is: Ben Maamar M, Lesné L, Desdoits-Lethimonier C, Coiffec I, Lassurguère J, et al. (2015) An Investigation of the Endocrine-Disruptive Effects of Bisphenol A in Human and Rat Fetal Testes. PLoS ONE 10(2): e0117226. doi:10.1371/journal.pone.0117226
